# Profil pressionnel de l’adolescent en milieu scolaire à Lubumbashi, République Démocratique du Congo

**DOI:** 10.11604/pamj.2018.29.94.14537

**Published:** 2018-01-31

**Authors:** Placide Kambola Kakoma, Emmanuel Kiyana Muyumba, Clarence Kaut Mukeng, Jaques Mbaz Musung, Christian Ngama Kakisingi, Olivier Mukuku, Dophra Ngoy Nkulu

**Affiliations:** 1 Département de Médecine Interne, Université de Lubumbashi, République Démocratique du Congo; 2 Département de Santé Publique, Université de Lubumbashi, République Démocratique du Congo; 3 Institut Supérieur des Techniques Médicales de Lubumbashi, République Démocratique du Congo

**Keywords:** Pression artérielle, paramètres anthropométriques, fréquence cardiaque, adolescence, Lubumbashi, Blood pressure, anthropometric parameters, heart rate, adolescence, Lubumbashi

## Abstract

**Introduction:**

L'objectif de cette étude était de donner le profil de la pression artérielle (PA) des adolescents âgés de 15 à 19 ans en milieu scolaire à Lubumbashi, République Démocratique du Congo.

**Méthodes:**

il s'agit d'une étude transversale, portant sur les adolescents âgés de 15 à 19 ans au moyen d'un échantillonnage aléatoire des écoles secondaires de Lubumbashi durant les années scolaires 2013-2014, 2014-2015 et 2015-2016. Trois mesures de PA étaient effectuées le même jour.

**Résultats:**

1766 adolescents âgés de 15-19 ans ont été inclus parmi eux 995 étaient de sexe féminin et 771 garçons. Les garçons avaient significativement une pression artérielle systolique élevée que les filles dans les tranches d'âges de 17, 18 et 19 ans. La pression artérielle diastolique n'était pas différente statistiquement dans toutes les tranches d'âges dans les deux sexes. Par contre, dans les deux sexes, la pression artérielle systolique été en corrélation significative avec le poids, la taille, l'indice de masse corporelle, le tour de taille et la fréquence cardiaque. Quant à la pression artérielle diastolique, des corrélations significatives étaient retrouvées avec le poids et l'indice de masse corporelle chez les filles alors que la fréquence cardiaque était en corrélation significative dans les deux sexes.

**Discussion:**

Au cours de notre étude, il était question de déterminer les valeurs moyennes de PA et sa corrélation avec les paramètres anthropométriques, la FC et le poids de naissance chez les adolescents d'âge compris entre 15 et 19 ans. Notre étude a révélé des valeurs moyennes de PAS chez les garçons qui étaient plus élevées que les filles statistiquement significatives dans les tranches d'âges de 17, 18 et 19 ans alors que les valeurs moyennes de PAD n'avait pas de différence statistiquement significative dans toutes les tranches d'âges dans les deux sexes. Harrabi et al. [16], dans leur étude incluant 1569 sujets âgés de 13 à 19 ans, avaient trouvé que les garçons de 16, 17 et 18 ans avaient des PAS élevées sans différence statistiquement significatives mais les différences statistiquement significatives étaient remarquées chez les filles de 13 et 14 ans concernant la PAD. Dans son étude chez les enfants, Forrester et al. [17] avaient rapporté une corrélation positive entre la PAS et l'âge chez les garçons et négative chez les filles. Cette corrélation négative trouvée entre la PAS et l'âge chez les filles pourrait être expliquée par les modifications hormonales liées à la puberté qui commencent plus tôt chez les filles que chez les garçons. Se référant à la littérature, la PA augmente avec la croissance en âge plus chez les garçons suite à l'augmentation de la masse musculaire pendant la puberté [18-20].

Notre étude a montré que la PAS était en corrélation significative avec le poids, la taille, l'IMC, le tour de taille et la FC dans les deux sexes. Ce constat est similaire à celui faite par l'étude de Harrabi et al. [16] qui rapportait que la PAS était en corrélation positive avec la taille (garçons: r = 0,33; p < 0,0001; filles: r = 0,08; p = 0,02), le poids (garçons: r = 0,47, p < 0,0001; filles: r = 0,35; p < 0,0001) et l'âge (r = 0,12; p < 0,0001). Quant à la PAD dans notre étude, les corrélations significatives positives étaient retrouvées avec le poids (r = 0,093; p = 0,003) et l'IMC (r = 0,079; p = 0,012) seulement chez les filles, alors que la FC était en corrélation significative positive chez les garçons (r = 0,168; p < 0,0001) mais non chez les filles (r = 0,12, p < 0,0001) [16]. Dans une étude similaire chez les adolescents réalisée par Sinaiko et al. [21], une corrélation a été trouvée entre le poids et la PAS chez les garçons (r = 0,167, p < 0,0001) et les filles (r = 0,112, p < 0,0001). L'effet de la taille et du poids sur la PA a déjà été démontré dans plusieurs études transversales antérieures sur les enfants concluant en une forte corrélation positive [22,23]. L'insuffisance de déclaration des naissances à l'état civil dans plusieurs pays en développement, conséquente au recours aux poids de naissance déclarés auprès des parents ou tuteurs, serait un biais dans la réalisation des résultats statistiquement comparables.

**Conclusion:**

Malgré les faiblesses potentielles de la présente étude dans sa conception transversale et les mesures de la PA le même jour, les données pourraient aider les responsables de la santé à adopter une stratégie nationale de prévention de l'hypertension artérielle dans notre population.

## Abstract (en)

**Introduction:**

This study aimed to highlight blood pressure (BP) profile in adolescents aged 15-19 years at school in Lubumbashi, Democratic Republic of the Congo.

**Methods:**

We conducted a cross-sectional study of adolescents aged 15-19 years through random sampling of the secondary schools in Lubumbashi during the school years 2013-14, 2014-15 and 2015-16. Blood pressure was measured three times in the same day.

**Results:**

Our study included 1766 adolescents aged 15-19 years, of whom 995 were females and 771 boys. Boys’ systolic blood pressure was significantly higher than that of girls aged 17, 18 and 19 years. Diastolic blood pressure was not statistically different in all different age groups in both sexes. On the other hand, in both sexes, systolic blood pressure was significantly correlated with body weight, size, body mass index, waist size and heart rate. With regard to diastolic blood pressure, significant correlations were found with body weight and body mass index in girls while heart rate was significantly correlated in both sexes.

**Conclusion:**

Despite the potential weaknesses of the present study with special regard to its cross-sectional design and to BP measures in the same day, collected data could help health authorities to adopt a national hypertension prevention strategy.

## Introduction

La pression artérielle (PA) est dite élevée (c'est-à-dire la pré-hypertension ou normale haute), lorsque la pression artérielle systolique (PAS) et/ou la pression artérielle diastolique (PAD) est ≥ 90^ème^ percentile mais < 95^ème^ percentile ou lorsque la PAS/PAD mesurée ≥120/80 mmHg pour le sexe, l'âge et la taille [1-4]. Elle doit attirer plus d'attention à cause de sa progression vers l'hypertension artérielle (HTA) à l'âge adulte [5] et de son association avec des atteintes viscérales infracliniques telles que l'augmentation de la masse ventriculaire gauche, la dysfonction diastolique [6], la présence de stries lipidiques et de plaques d'athérome dans les artères coronaires et l'aorte [7] et à l'épaississement des parois artérielles [8] et elle est l'une des principales causes de décès et d'incapacité dans le monde [9]. Toutefois, les informations démographiques de niveaux de PA chez les enfants et les adolescents en fonction de l'âge, du sexe et la taille ont déjà été réalisées dans d'autres pays à travers le monde et ont attiré notre attention. De ce qui précède et compte tenu du fait qu'à notre connaissance, il n'existe pas dans notre milieu des études basées sur la mesure des valeurs de la PA et sa corrélation avec les paramètres anthropométriques, la fréquence cardiaque (FC) et le poids de naissance chez les adolescents d'âge compris entre 15 et 19 ans, ceci motive la présente étude. La présente étude s'est fixée comme objectif de déterminer les valeurs moyennes de PA et sa corrélation avec les paramètres anthropométriques, la FC et le poids de naissance chez les adolescents d'âge compris entre 15 et 19 ans.

## Méthodes

### Type et population et période d'étude

Il s'agit d'une étude transversale menée dans une population constituée d'adolescents âgés de 15 à 19 ans, régulièrement inscrits dans les écoles ciblées de Lubumbashi (chef-lieu de la Province du Haut-Katanga et deuxième grande ville de la République Démocratique du Congo) dont le parent ou le tuteur a signé librement le consentement éclairé durant les années scolaires 2013-2014, 2014-2015 et 2015-2016.

### Echantillonnage

Notre échantillon était composé de 1766 adolescents. Les élèves été recrutés de manière aléatoire selon un plan d'échantillonnage stratifié en grappes à deux niveaux. Ainsi, ont été sélectionnés successivement un échantillon d'écoles (réparties par commune), puis un échantillon des classes dans chaque école sélectionnée, enfin tous les élèves (unités statistiques) inscrits à l'intérieur des classes sélectionnées étaient retenus comme adhérant à l'enquête. Les données ont été recueillies par l´équipe médicale d´étude formée qui recevait une formation de recyclage au début de chaque phase de collecte de données.

### Critères d'inclusion

Ont été inclus dans notre étude: être âgé de 15 à 19 ans et régulièrement inscrit dans une école primaire ou un établissement secondaire de la ville de Lubumbashi; Avoir un poids de naissance déclaré par l'un des parents ou le tuteur légal de l'élève ayant consenti de subir l´examen sans contrainte; Parents/tuteur ayant signé le consentement éclairé écrit, selon la déclaration d'Helsinki et ses amendements avant la participation à l'étude.

### Critères de non inclusion

Ont été exclus de cette étude: tout élève ne remplissant pas les conditions de scolarisation ou d´âge; Elève ou parent ayant manifesté un refus de subir l´examen; Maladie chronique qui influence PA: néphropathie, diabète sucré, ATCD personnel HTA; Etre sous traitement médicamenteux qui peut influencer la pression artérielle: Antihypertenseurs, anti arythmiques, psychostimulants, corticothérapie, hormones thyroïdiennes.

### Variables d'étude

Les variables de notre étude étaient: l'âge, le sexe, la PAS, la PAD, le poids, la taille, l'indice de masse corporelle, le Tour de taille, la fréquence cardiaque (FC) et le poids de naissance (PN).

La PA et la fréquence cardiaque (FC) ont été mesurées à l'aide d'un tensiomètre oscillométrique automatique de marque Datascope Accutorr Plus (Datascope Corporation, USA). Les mesures de la PAS et de la PAD étaient établies par la moyenne des deux dernières des trois mesures, et étaient considérées pour l'analyse. Cet appareil donnait au même moment les chiffres pressionnels et la FC. Les brassards de taille appropriée, de marque Critikon (GE Healthcare, USA) ont également été utilisés, en fonction de la largeur du bras (au moins 40% de la circonférence du bras) et de sa longueur (12 x 19 cm, 17 x 25 cm, 23 x 33 cm, pour couvrir 80-100% de la circonférence du bras de l'individu). La taille du brassard était déterminée en mesurant la circonférence à la mi-hauteur du bras. Les mesures de la PA et de la FC étaient effectuées, au moins 30 minutes après l'exercice physique ou le dernier repas, chez un sujet au repos de 5 minutes avant la prise [10-12]. Le sujet était dans une position assise avec le bras et le dos soutenus, le brassard au niveau du cœur, pieds reposant sur le plancher et les jambes non croisées. Le brassard était appliqué au bras droit, puis enveloppé à une étanchéité qui ne permettait pas à deux doigts d´être insérés sous celui-ci. Le bord inférieur du brassard était placé à 2 cm de la fossette cubitale. Chaque élève était soumis à trois mesures de la PA et de la FC, à une minute d'intervalle, le même jour. Le poids (exprimé en kg enregistré à 0,1 kg près) était mesuré à deux reprises, chez un sujet légèrement habillé, déchaussé, débout sur une balance médicale numérique de marque SECA (SECA 881 U, Allemagne).

La taille, enregistrée à 0,1 centimètre près, était mesurée à deux reprises à l'aide d'une toise verticale, chez un sujet déchaussé, debout, avec les épaules et les hanches perpendiculaires à l'axe central, les talons contre le marchepied, les genoux joints, les bras relâchés le long du corps et la tête bien droite (tête regardant devant de façon à ce que le rebord inferieur des orbites soit dans le même plan horizontal que le conduit auditif externe). Le tour de taille, selon le protocole de National Institutes of Health [10,13], a été mesuré à deux reprises, à l'aide d'un mètre ruban non extensible, directement sur la peau, au niveau des crêtes iliaques, au moment de l'expiration, chez un sujet débout, relaxé, ne rentrant pas son ventre. L'IMC était calculé à partir de la formule: IMC = poids (en kilogrammes) divisé par taille (en mètres) au carré et exprimé en kg/m². Tenant compte de l'insuffisance de déclaration des naissances à l'état-civil dans notre pays comme dans d'autres en développement, notre étude avait recouru aux poids de naissance déclarés auprès des parents ou tuteurs, stratégie qu'avait adoptée l'UNICEF et l'OMS en 2004 pour l'estimation du faible poids de naissance dans les pays en développement [14].

### Gestion des données et analyses statistiques

Les données ont été saisies à l'aide du logiciel Epi Info version 3.5.1 et analysées au moyen du logiciel SPSS 21.0. Les variables quantitatives étaient représentées sous forme de moyenne, d'écart-type (ET), d'intervalle de confiance à 95% (IC95%) et alors que celles qualitatives sous forme d'effectifs (n) et pourcentage (%). Pour faciliter les calculs, l'âge était décimalisé au moyen de la formule d'Ireton [15]. Le test t de Student de comparaison de moyennes de l'âge, du sexe, de la taille, de la FC, du tour de taille, de l'IMC, du poids de naissance, de la PAD et de la PAD. Le test de Khi-carré de Pearson a été utilisé pour évaluer l'association entre variables qualitatives. La valeur de p < 0,05 était considérée comme statistiquement significative et un test bilatéral a été utilisé. Les moyennes des deux dernières des trois mesures de la PAS, de la PAD et de la FC étaient établies et considérées pour l'analyse.

### Considérations éthiques

Le consentement éclairé par écrit était obtenu auprès du parent ou du tuteur de l'adolescent enquêté. L'approbation de la tenue de l'étude ainsi que les autorisations y afférentes étaient obtenues du Comité d'Ethique Médicale de l'Université de Lubumbashi, du Ministère Provincial de l'Education du Katanga et des responsables des écoles sélectionnées.

## Résultats

L'étude avait porté sur 1766 adolescents dont 995 filles (56,3%) et 771 garçons (43,7%) âgés de 15 à 19 ans. En comparant les deux sexes, nous constatons que les moyennes (ET) des variables suivantes étaient statistiquement différentes (p < 0,0001): les moyennes du poids, de la taille, de l'IMC, de la PAS et de la FC étaient respectivement de 55,6 (8,0) kg, de 166 (9,3) cm, de 20,2 (2,4)kg/m^2^, de 118,5 (11,3) mmHg et de 74 (12,3) battements/minute chez les garçons contre 53,5 (7,7)kg, 159,1 (8,1)cm, 21,2 (3,2)kg /m^2^, 116,1 (10,3)mmHg et 83,1 (11,8) battements/minute chez les filles. Quant au tour de taille, à la PAD et au PN, les valeurs des moyennes n'avaient pas été statistiquement différentes entre les deux sexes (p > 0,05) (Tableau 1). Dans les deux sexes, la PAS était en corrélation significative avec le poids (garçons: r = 0,271, p < 0,0001; filles: r = 0,186, p < 0,0001), la taille (garçons: r = 0,177, p < 0,0001; filles: r = 0,110, p < 0,0001), l'IMC (garçons: r = 0,178, p < 0,0001; filles: r = 0,111, p < 0,0001), le tour de taille (garçons: r = 0,220, p = 0,000; filles: r = 0,118, p < 0,0001) et la FC (garçons: r = 0,085, p = 0,018; filles (r = 0,324, p < 0,0001). Quant à la PAD, des corrélations significatives étaient retrouvées avec le poids (r = 0,093, p = 0,003) et l'IMC (r = 0,079, p = 0,012) seulement chez les filles; la FC était en corrélation significative avec la PAD chez les garçons (r = 0,168, p < 0,0001) et chez les filles (r = 0,237, p =< 0,0001) (Tableau 2).

**Tableau 1:** Caractéristiques générales des 1766 adolescents selon le sexe et les groupes d’âge de 15-17 et 18-19 ans à Lubumbashi, RDC

	Groupes d’âge (en années)			
**Sujets inclus, n**	15 -17	18 - 19	Total (n, ET)	%
Garçons	518	253	771	43,7
Filles	771	224	995	56,3
**Variables**				*p*
Poids, moyenne (DS) en Kg				
Garçons	54,2(7,9)	58,4 (7,4)	55,6 (8)	<0,0001
Filles	52,9 (7,7)	55,4 (7,4)	53,5 (7,7)	
**Taille, moyenne (DS) en Cm**				
Garçons	164,9(9,2)	168,4(9,1)	166(9,3)	<0,0001
Filles	159(7,9)	159,3(8,8)	159,1(8,1)	<0,0001
**Tour de Taille, moyenne (DS) en cm**				
Garçons	70,2(4,8)	72,3(5,2)	70,9(5)	0,2300
Filles	70,8(4,8)	72,5(6,7)	71,1(6,2)	
**IMC, moyenne (DS) en Kg/m^2^**				
Garçons	19,9(2,4)	20,6(2,3)	20,6(2,3)	<0,0001
Filles	21(3,1)	22(3,4)	21,2(3,2)	
**PAS, moyenne (DS), en mmHg**				
Garçons	117,9(11,2)	119,8(11,3)	118,5(11,3)	<0,0001
Filles	116,2(10,3)	115,7(10,4)	116,1(10,3)	
**PAD, moyenne (DS) en mmHg**				
Garçons	68(8,2)	68,8(8)	68,3(8,2)	0,211
Filles	68,3(7,6)	69,5(8,2)	68,6(7,8)	
**FC, moyenne (DS) en battement/min**				
Garçons	74,9(12,2)	72(12,3)	74(12,3)	<0,0001
Filles	83,5(11,6)	81,7(12,2)	83,1(11,8)	
**PN, moyenne (DS) en gramme**				
Garçons	3250,35(566,0)	3352,7(569,6)	3296,4(674,2)	0,012
Filles	3216,6(575,8)	3206,1(598,8)	3214,3(580,8)	

n: effectif ; DS : déviation standard; IMC: Indice de masse corporelle; Batt/min: Battement par minute; PN: poids de naissance

**Tableau 2:** Corrélation PAS et PAD et les paramètres anthropométriques, FC et PN chez 1766 adolescents de 15 à 19 ans (771 garçons et 995 filles) selon le sexe à Lubumbashi, RDC

	Sexe			
Variable	Garçons		Filles	
	r	p	r	p
PAS				
Poids	0,271**	0,000	0,186**	<0,0001
Taille	0,177**	0,000	0,110**	0,001
IMC	0,178**	0,000	0,111**	<0,0001
TT	0,220**	0,000	0,118**	<0,0001
FC	0,085*	0,018	0,324**	<0,0001
PN	0,031	0,476	-0,002	0,967
PAD				
Poids	0,070	0,051	0,093**	0,003
Taille	0,034	0,347	0,027	0,395
IMC	0,058	0,109	0,079*	0,012
TT	0,067	0,065	0,033	0,294
FC	0,168**	0,000	0,237**	<0,0001
PN	0,023	0,593	-0,008	0,831

PAS, pression artérielle systolique. PAD, pression artérielle diastolique. IMC, indice de masse corporelle. TT, tour de taille. FC, fréquence cardiaque. PN, poids de naissance, r, coefficient de corrélation de Pearson. *p*<0,05.

** Corrélation significative au niveau 0,01.

* Corrélation significative au niveau 0,05

## Discussion

Au cours de notre étude, il était question de déterminer les valeurs moyennes de PA et sa corrélation avec les paramètres anthropométriques, la FC et le poids de naissance chez les adolescents d'âge compris entre 15 et 19 ans. Notre étude a révélé des valeurs moyennes de PAS chez les garçons qui étaient plus élevées que les filles statistiquement significatives dans les tranches d'âges de 17, 18 et 19 ans alors que les valeurs moyennes de PAD n'avait pas de différence statistiquement significative dans toutes les tranches d'âges dans les deux sexes. Harrabi et al. [16], dans leur étude incluant 1569 sujets âgés de 13 à 19 ans, avaient trouvé que les garçons de 16, 17 et 18 ans avaient des PAS élevées sans différence statistiquement significatives mais les différences statistiquement significatives étaient remarquées chez les filles de 13 et 14 ans concernant la PAD. Dans son étude chez les enfants, Forrester et al. [17] avaient rapporté une corrélation positive entre la PAS et l'âge chez les garçons et négative chez les filles. Cette corrélation négative trouvée entre la PAS et l'âge chez les filles pourrait être expliquée par les modifications hormonales liées à la puberté qui commencent plus tôt chez les filles que chez les garçons. Se référant à la littérature, la PA augmente avec la croissance en âge plus chez les garçons suite à l'augmentation de la masse musculaire pendant la puberté [18-20].

Notre étude a montré que la PAS était en corrélation significative avec le poids, la taille, l'IMC, le tour de taille et la FC dans les deux sexes. Ce constat est similaire à celui faite par l'étude de Harrabi et al. [16] qui rapportait que la PAS était en corrélation positive avec la taille (garçons: r = 0,33; p < 0,0001; filles: r = 0,08; p = 0,02), le poids (garçons: r = 0,47, p < 0,0001; filles: r = 0,35; p < 0,0001) et l'âge (r = 0,12; p < 0,0001). Quant à la PAD dans notre étude, les corrélations significatives positives étaient retrouvées avec le poids (r = 0,093; p = 0,003) et l'IMC (r = 0,079; p = 0,012) seulement chez les filles, alors que la FC était en corrélation significative positive chez les garçons (r = 0,168; p < 0,0001) mais non chez les filles (r = 0,12, p < 0,0001) [16]. Dans une étude similaire chez les adolescents réalisée par Sinaiko et al. [21], une corrélation a été trouvée entre le poids et la PAS chez les garçons (r = 0,167, p < 0,0001) et les filles (r = 0,112, p < 0,0001). L'effet de la taille et du poids sur la PA a déjà été démontré dans plusieurs études transversales antérieures sur les enfants concluant en une forte corrélation positive [22,23]. L'insuffisance de déclaration des naissances à l'état civil dans plusieurs pays en développement, conséquente au recours aux poids de naissance déclarés auprès des parents ou tuteurs, serait un biais dans la réalisation des résultats statistiquement comparables.

## Conclusion

Dans un pays en développement face à la transition épidémiologique comme la RDC, il semble logique que la prévention des maladies cardiovasculaires commence dans l'enfance. Malgré les faiblesses potentielles de la présente étude, comme sa conception transversale et la mesure unique de la PA, les données aideront les responsables de la santé à adopter une stratégie nationale pour prévenir l'extension de l'HTA dans la population.

### Etat des connaissances actuelle sur le sujet

- La pression artérielle (PA) élevée est une importante cause d'incapacité et de décès dans le monde;
- La PA élevée à un jeune âge est un facteur de risque d'hypertension à l'âge adulte;
- Le lien avec la PA augmente avec l'indice de masse corporelle.

### Contribution de notre étude à la connaissance

- Les garçons ont une pression artérielle systolique élevée que les filles dans les tranches d'âges de 17, 18 et 19 ans;
- La pression artérielle systolique est en corrélation avec le poids, la taille, l'indice de masse corporelle, le tour de taille et la fréquence cardiaque;
- La pression artérielle diastolique est corrélée avec le poids et l'indice de masse corporelle chez les filles.

## Conflits d’intérêts

Les auteurs ne déclarent aucun conflits d''intérêts.
